# Effect of Pod Storage and Drying Temperature on Fermentation Dynamics and Final Bean Quality of Cacao Nacional in Ecuador

**DOI:** 10.3390/foods13101536

**Published:** 2024-05-15

**Authors:** Stefanie Streule, Susette Freimüller Leischtfeld, Karin Chatelain, Susanne Miescher Schwenninger

**Affiliations:** ZHAW Zurich University of Applied Sciences, Institute of Food and Beverage Innovation, 8820 Wädenswil, Switzerland; stefanie.streule@zhaw.ch (S.S.); susette.freimueller@zhaw.ch (S.F.L.); karin.chatelain@zhaw.ch (K.C.)

**Keywords:** cocoa beans, pod-opening delay, post-harvesting, quality assessment, sensory analysis

## Abstract

The impact of pod storage (PS) and two drying temperatures of fermented cocoa beans was investigated in Ecuador. Therefore, four variations were simultaneously carried out three times at two locations, independently: 0, 3, and 5 days of PS, dried at 60 °C and 0 days of PS, dried at 80 °C. Pod weight during storage, pulp content, pH, temperature, microbial counts, total free amino acids, protein profiles, sugars, organic acids, cut-test, fermentation index, and sensory profiles were analyzed. Minor differences in fermentation dynamics and bean quality were found between variations with and without PS. A rather accelerated fermentation with pod-stored beans was observed (e.g., faster color change, slightly lower pH in cotyledon after 48 h), along with a significantly higher maximal temperature during 24–42 h (43.1 ± 3.2 °C compared to 39.2 ± 2.0 °C without PS). More well-fermented beans were reached with PS (52.3 ± 22.6%) than without (62.7 ± 9.2%). Differences during fermentation were observed between the locations (e.g., pH, acids, sugars), but sensory evaluation indicated that the impact of location was mitigated with PS. Drying at 80 °C showed no adverse effects, as evidenced by the results of the cut-test and fermentation index. However, sensory evaluations revealed significant differences between 80 °C and 60 °C, with the former exhibiting more bitter and astringent cocoa liquor.

## 1. Introduction

Cacao Nacional trees, renowned for their fine flavor, constitute one of Ecuador’s varieties and account for 63% of global fine flavor production [1]. Despite its prominence in quality cocoa production, Ecuador ranks third among cocoa-producing countries worldwide [1,2,3,4]. Cocoa beans undergo processing in their country of origin before being shipped to chocolate-producing countries [5]. The post-harvesting process, which includes fermentation and drying, plays a crucial role in determining cocoa bean quality by facilitating the development of flavor precursors and reducing moisture content for safe storage and shipment [6,7,8]. However, diverse practices for post-harvesting processes can be found [4,6], often carried out by small agricultural producers, leading to variable quality [8,9].

Cocoa bean quality, including factors like acidity, aromatic profile, and cut-test results, can be intentionally enhanced through various methodologies explored in past research. Quality can be improved by standardized post-harvesting processes, which starts with the selection of an adequate fermentation device (e.g., boxes, bags). Fermentation should have the right duration, possibly including turning steps during the process [6,10,11,12,13,14,15,16,17,18,19,20]. Additionally, the application of functional microbial cultures has shown promise in accelerating fermentation processes, enhancing sensory profiles, and preventing fungal growth, among others [19,21,22,23,24,25,26,27,28]. Furthermore, various approaches to pulp pre-conditioning have been documented in the literature, including pod storage, mechanical or enzymatic depulping, and pre-drying [29,30,31]. The practice of pod storage is commonly applied and investigated in Malaysia, Ghana, and the Ivory Coast, where cacao pods are collected after harvesting for a period before fermentation of the beans [13,31,32,33,34,35]. Conversely, pre-drying, often observed in countries like Ecuador, includes spreading beans on a concrete floor before fermenting them in, for example, jute bags [6,16]. Pre-conditioning of the pulp aims to reduce acidity, accelerate the fermentation process, and enhance the quality, such as the flavor profile or cut-test [20,30,31,33,36,37]. Furthermore, it is possible to mechanically remove a portion of the cocoa pulp from the beans after pod opening without negatively affecting the quality, as not all of the cocoa pulp is required for the fermentation process [29,38,39]. This valorizes the complete cacao fruit by utilizing its high-value byproducts for other purposes [29,40].

The post-harvesting process also includes drying the cocoa beans after fermentation, reducing their moisture content from about 60% to approximately 7% [41]. During drying, further chemical changes occur, contributing to the development of flavor through the formation of volatile and non-volatile compounds [41,42,43]. Cocoa drying methods typically include sun drying or artificial drying. In regions where cacao is harvested all year round, artificial drying becomes necessary, especially during the wet seasons to prevent mold contamination [41]. Artificial drying methods can be influenced by the choice of dryer and adjustment of drying temperatures [6,43]. To date, the influence of the drying techniques and conditions on cocoa quality have already been studied. During artificial drying, processing temperatures of more than 60 °C are often applied and can lead to the risk of having increased free fatty acid and acetic acid levels and, therefore, a decreased pH in dried cocoa beans [44,45]. Additionally, direct contact with fire in dryers without ventilators can lead to temperatures as high as 95.2 ± 13.7 °C, causing roasted/burnt off-flavors in dried beans [6,16]. Studies have shown that drying temperatures of 70–80 °C yield similar volatile profiles to sun-drying, with no significant differences in key compounds such as alcohols, aldehydes, ketones, esters, acids, and pyrazines. Choosing lower temperatures also offers the advantage of reducing drying costs [43].

To the best of our knowledge, in the context of Ecuadorian Cacao Nacional, the impact of pod storage on fermentation and cocoa quality remains understudied, despite its common practice among some farmers. Expanding on prior research by Streule et al. [6,16], our study introduces an examination of two specific drying temperatures, utilizing identical dryers, to uncover their effects on cocoa bean quality. Conducted concurrently at two locations, our research tracks the post-harvesting process, including different pod storage times, standardized fermentation, and drying at different temperatures, while analyzing parameters such as pod weight during storage, pulp content at the beginning of fermentation, pH, temperature, microbial counts, total free amino acids and changes in protein profiles, sugars and organic acids, cut-tests, fermentation index, and sensory profiles. By elucidating the influence of pod storage and drying temperatures on Ecuadorian cocoa quality, our findings aim at understanding the potential of optimized cocoa production practices, ultimately enhancing cocoa bean quality.

## 2. Materials and Methods

### 2.1. Set-Up Experiments and Sampling

**Figure 1:**
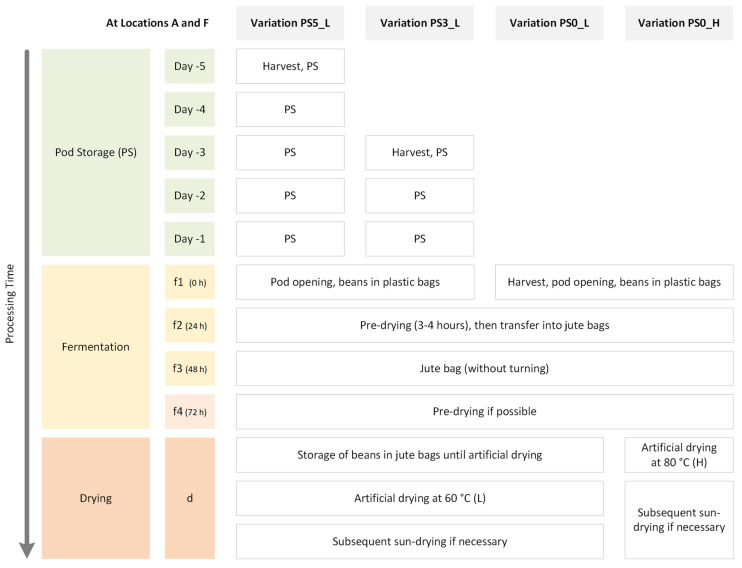
Set-up of post-harvest experiments with four variations carried out simultaneously: PS5_L and PS3_L with previous pod storage and simultaneous fermentation start on f1 alongside variations PS0_L and PS0_H, where fermented beans were dried at 60 °C and 80 °C, respectively. At each location A and F, three independent runs within this experimental framework were executed.

The experiments were carried out between September 2021 and November 2021 with the Cacao Nacional variety (selected as described by Streule et al. [6]) in Manabí (A) and Guayas (F) in Ecuador. In this publication, “cacao” refers to the variety, tree, and pods, whereas “cocoa” is utilized for the beans once they are processed, starting from their extraction of the pods. The experiments (Figure 1) were carried out in three independent runs, investigating, on the one hand, the influence of the pod storage (PS) (subjected to a low drying temperature of 60 °C) and, on the other hand, high (80 °C) and low (60 °C) drying temperatures (without undergoing pod storage).

**Figure 2:**
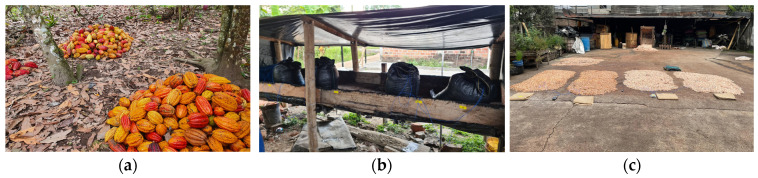
(**a**) Pile of pods during pod storage at location A; (**b**) fermentation beginning in plastic bags at location F; (**c**) pre-drying on day 1 on concrete floor at location A.

For the variations PS5_L and PS3_L, where pod storage was performed, ripe and healthy pods were harvested (approx. 500 pods per run and variation) without damaging the pods. Pods were then piled on the ground under cacao trees, protected from the rain and sun for 5 (PS5_L) or 3 (PS3_L) days (Figure 2a). Pods for variations PS0_L/PS0_H were harvested likewise on the same day as the fermentation started.

Pods were opened on day f1 and the beans were extracted. About 45–55 kg healthy beans, without placenta, were put in permeable plastic bags which were placed next to each other on the concrete floor (location A) or on a covered wooden shelf (location F, Figure 2b) for draining overnight. At location A, the bags were covered with a plastic cover during the night.

For the pre-drying (day f2), the beans were spread in a layer of a height of about four beans on the concrete floor (Figure 2c) and pre-dried for 3–4 h with turnings every 30 min. Then, the beans were put in separate jute bags (volume 63 kg of dried cocoa beans) and placed as the day before. During the night, the jute bags were protected from moisture with a plastic cover (location A) or left on the covered wooden shelf (location F). Bags were left until f4 when drying started. Beans were sun-dried first on the concrete floor for 0–7.5 h (no pre-drying possible during run 2 at location A due to rainy weather) with turnings every 30 min.

**Figure 3:**
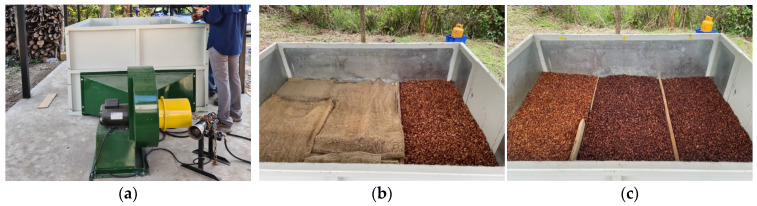
(**a**) Artificial dryer SIRCA (Model: SR-10-SI) at location F; (**b**) beans from variation PS0_H in 1/3 of dryer and rest covered with jute bags at location A; (**c**) beans of variations PS5_L, PS3_L, PS0_L separated in dryer with wooden sticks at location A.

Then, the beans from variation PS0_H were dried artificially (SIRCA, Guayaquil, Ecuador, Model: SR-10-SI, capacity 400 kg, Figure 3a) for 4–8.5 h at 80 °C while spreading the beans in the whole dryer (run 1) or dried in just one third of the dryer’s effective area, leaving the remaining space empty and covered by two layers of jute bags (runs 2 and 3, Figure 3b). The other beans from variations PS5_L, PS3_L, and PS0_L were stored in jute bags until the next morning for the artificial drying (9–15 h at 60 °C, Figure 3c).

If the beans did not reach a moisture of <8% (mini GAC moisture tester, Dickey-John, Auburn-IL, USA) during artificial drying, they were sun-dried again on the concrete floor at a later time, after artificial drying (0–10 h).

Cocoa bean sampling during fermentation was performed by hand using disinfected gloves directly at the fermentation place. The beans were taken from different points according to the positions of the temperature probes. Microbial counts of fresh and fermenting beans, pulp and cotyledon pH of fresh, fermenting, and dried beans were analyzed directly afterwards. The cut-test was also carried out directly after drying on-site. Dried samples were sent to Switzerland for further analyses (sugars, organic acids, fermentation index, protein analysis, total free amino acids content, sensory evaluation; see Section 2.2.5, Section 2.2.6, Section 2.2.7, Section 2.2.8 and Section 2.2.9).

### 2.2. Analyses

#### 2.2.1. Monitoring of Pod Weight, Pod and Bean Color

**Figure 4:**
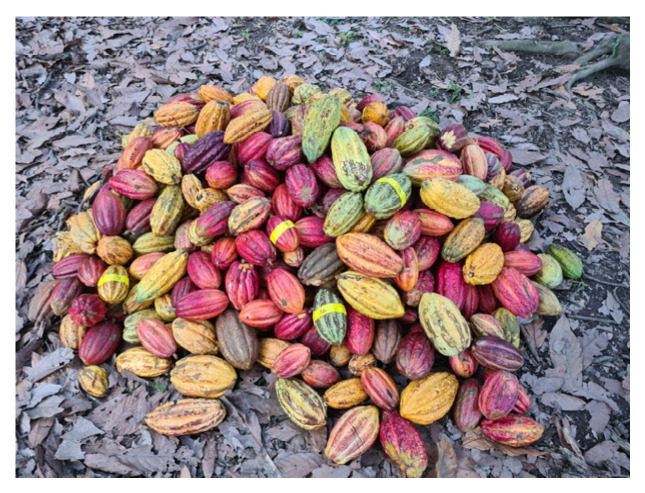
Selected pods from piles during pod storage, marked with yellow tape at location A.

After harvesting for variations PS5 and PS3, 10 pods were selected out of 500 and marked with tape (Figure 4). These fruits were stored randomly in the pile and monitored daily during pod storage (PS) of PS5_L and PS3_L. Each pod was weighed by an electronic kitchen scale and pod appearance (color) was observed during PS. Further, the bean color during fermentation was observed and described after sample taking. Pictures of pods and beans were taken daily.

#### 2.2.2. Measurement of Pulp Content, pH of Pulp and Cotyledon

The content of pulp on the first day of fermentation was determined, as described by Romanens et al. [46], where 20 cocoa beans were weighed before and after the pulp was removed using paper towels. The pH values were measured using indirect methods according to Romanens et al. [23,46]. Ten g of cocoa beans was manually kneaded with an equal amount of dH_2_O in a plastic bag for the pH pulp, while for the pH cotyledon, 20 g of beans was peeled, mixed with 180 g of dH_2_O using a blender (Bamix AG, Mettlen, Switzerland), both for 2 min. The pH from the pulp–water and cotyledon–water mixture was then measured (VWR International, Radnor, PA, USA).

#### 2.2.3. Measurement of Temperature

The temperature of the bean–pulp mass during fermentation was recorded every 15 min with a datalogger (testo, 176T4, Testo AG, Mönchaltdorf, Switzerland). Three probes per variation were positioned in the bags (two probes in the middle of the bag, one of which was placed in the upper layer, and the third probe towards the edge of the bag), according to the method of Streule et al. [6], during fermentation.

#### 2.2.4. Enumeration of Yeasts and Lactic Acid Bacteria

Yeasts and lactic acid bacteria (LAB) were determined daily and twice per variation and run during fermentation. Ten g of the sample was mixed with dilution solution and a serial dilution was prepared as described by Streule et al. [16]. One ml of the dilutions was then surface plated on 3M™ Petrifilm^®^ Rapid Yeast and Mold Count Plates for yeasts and on 3M™ Petrifilm^®^ Lactic Acid Bacteria Count Plates for LAB (both from 3M Food Safety, St. Paul, MN, USA). All plates were incubated at room temperature for 2 to 4 days.

#### 2.2.5. Sugars and Organic Acids by HPLC

Saccharose, glucose, fructose, citric acid, lactic acid, and acetic acid were determined in all cotyledon samples after drying. The samples were prepared as described by Romanens et al. [23,46] with the modification of using the cotyledons without shells and adjusting the centrifugation parameters to 15 min at 17,000× *g* at 4 °C. High-performance liquid chromatography (HPLC) was performed on an HPLC system (Agilent 1260, Santa Clara, CA, USA), while using a ROA–Organic Acid H+ column for sucrose, glucose, citric acid, lactic acid, acetic acid, and Rezex RPM monosaccharides for fructose. A Refractive Index (RI) detector (1260 RID G1362A, Agilent, Santa Clara, CA, USA, temperature set to 50 °C) was utilized for sugars and a Diode Array Detector (DAD) (1260 RID G4212B, Agilent, Santa Clara, CA, USA, wavelength set to 210 nm) for organic acid analyses [16].

#### 2.2.6. Cut-Test and Fermentation Index (FI) of Dried Beans

The cut-test was performed per variation by cutting 3 × 100 beans lengthwise to expose a maximum cotyledon surface, classifying the cut beans by the following attributes: well-fermented (brown color on the complete surface), slightly fermented (brownish with violet parts), violet (violet or purple color on at least half of the surface), slaty (slaty color on at least half of the surface), and moldy (mold visible to the naked eye) beans, based on Streule et al. [6] and ISO 2451:2017 [47].

FI was determined using the method described by Gourieva and Tserrevitinov [48] with minor modifications. For this, 250 mg cocoa powder (frozen cocoa nibs were mixed for 30 s with a hand blender (Bamix AG, Mettlen, Switzerland)) was homogenized with 25 mL methanol/HCl (97:3 (*v*/*v*)) solution. This mixture was left for 17 h at 5 °C prior to filtering through a Whatman filter paper. The absorbance was read three times at wavelengths 460 nm and 530 nm (Synergy HTX, BioTek Instruments, Winooski, VT, USA) and the ratio of 460 nm to 530 nm was calculated as FI.

#### 2.2.7. Protein Analysis by Sodium Dodecyl Sulfate Polyacrylamide Gel Electrophoresis (SDS-PAGE)

Cocoa bean samples were collected at various stages: f1 (including all samples from location A and those from location F collected during runs 1 and 3), f3 (samples from run 1 at both locations), f4 (all samples), and dEnd (dried beans, all samples) were analyzed.

All chemicals and reagents were supplied by Carl Roth AG, Arlesheim, Switzerland (30% acrylamide/bis (Rotiphorese ^®^ Gel 30 (37,5:1), SDS (sodium dodecyl sulfate), TEMED (N,N,N′,N′-Tetramethylethylendiamin), APS (Ammonium peroxydisulphate), 2-propanol), Sigma-Aldrich, Zug, Switzerland (tris base, HCl fuming 37%, DTT (DL-Dithiothreitol), BSA (Bovine serum Albumin), acetic acid), Thermo Fisher Scientific AG, Reinach, Switzerland (ladder (PageRuler Plus Prestained Protein Ladder), bromphenol blue, PageBlue protein staining solution), AppliChem GmbH, Darmstadt, Germany (glycerol anhydrous, saccharose), and by VWR International GmbH, Dietikon, Switzerland (acetone).

For SDS-Page, proteins were extracted from cocoa beans following a modified method by Hansen et al. [49]. One gram of cocoa powder (see Section 2.2.6) was mixed with 10 mL of 80% acetone using a polytron (Kinematica AG, Malters, Switzerland, Type PT 2500 E) for 1 min at 10,000× *g* and then left to rest for 5–10 min before centrifugation at 15,000× *g* and 4 °C for 5 min. The supernatant was removed, and the precipitate was mixed again with 10 mL of 80% acetone, followed by centrifugation. This process was repeated 5 times. The same procedure was repeated twice with 100% acetone before drying the pellet to obtain the dried cocoa protein powder.

One hundred μg of the dried cocoa protein powder was mixed with 400 μL ddH_2_O. Five μL of the mixture was combined with 4 μL 2 × SDS-PAGE sample buffer (90 mM Tris base, 2% SDS, 0.02% bromophenol blue and 20% glycerol, pH 6.8) and 1 μL DTT, heated at 70 °C for 15 min and centrifuged (30 s with Mini centrifuge, Gilson, Emmen, Switzerland, Type: PMC-880). The positive control (BSA) serves as a reference to verify the accuracy of the SDS-Page procedure and was processed in a separate tube. Electrophoresis was carried out at 200 V for 40 min in 1 × running buffer (25 mM Tris base, 192 mM Glycine, 0.1% SDS) using the Mini-Protean chamber (Biorad) with a 4% (*v*/*v*) acrylamide/bis (37, 5:1) stacking gel [containing 3 mL ddH_2_O, 0.66 mL 30% acrylamide/bis (37, 5:1), 1.26 mL 0.5 M Tris-HCl pH 6.8, 50 μL SDS (10%), 5 μL TEMED, 25ul APS (10%)] and a 14% (*v*/*v*) acrylamide/bis (37, 5:1) resolving gel [containing 1.65 mL ddH_2_O, 4.7 mL 30% acrylamide/bis (37, 5:1), 2.5 mL 1.5 M Tris-HCl pH 8.8, 100 μL SDS (10%), 5 μL TEMED, 50 μL APS (10%), 1 mL glycerol anhydrous]. After polymerization, 5 μL Ladder and 10 μL of each prepared sample (supernatant) were loaded into the SDS-PAGE gel.

After electrophoresis, the gel was washed 3 × for 10 min with ddH_2_O on a platform shaker (Heidolph Instruments GmbH & CO., Schwabach, Germany, KG, Type: Polymax 1040). Subsequently, the gel was incubated for 15 min in an isopropanol/acetic acid solution (25% isopropanol, 10% acetic acid), followed by a 5 min ddH_2_O wash. The gel was then incubated in 20 mL of PageBlue protein staining solution for 30–60 min and washed with ddH_2_O for 20 min before visualization. Bands were visualized using a UV transillumination (Azure biosystems c300, Dublin, CA, USA).

#### 2.2.8. Spectrophotometric Determination of Total Free Amino Acids Content

The same samples as in Section 2.2.7 were analyzed, each with a technical replicate. A modified photometric method according to Church et al. [50] was used for the determination of the free amino acid content in the cocoa beans.

Sodium tetraborate was sourced from Thermo Fisher Scientific AG (Reinach, Switzerland), glycine and SDS (sodium dodecyl sulfate) from Carl Roth AG (Arlesheim, Switzerland), and β-mercaptoethanol, sodium acetate, and acetic acid from Sigma-Aldrich (Zug, Switzerland). O-Phthalaldehyde was obtained from MP Biomedicals (Lucerna Chem AG, Luzern, Switzerland), trichloroacetic acid from AppliChem GmbH (Darmstadt, Germany), and methanol from VWR International GmbH (Dietikon, Switzerland).

Blank and OPA reagent were prepared based on the method of Church et al. [50]. Blank reagent: The following reagents were prepared and mixed: in a 50 mL volumetric flask, 10 mL of a 50 g/L (*w*/*v*) SDS solution and 100 µL of β-mercaptoethanol were added and the flask was filled with a 10 g/L (*w*/*v*) solution of sodium tetraborate; OPA reagent: the OPA reagent was prepared by adding 1 mL of a 40 mg/mL (*w*/*v*) o-phthalaldehyde solution in methanol to the blank solution; Extraction solution according to Murthy et al. [51]: here, 0.11 mol/trichloroacetic acid (17.79 g/L), 0.22 mol/L sodium acetate (18.05 g/L), and 0.33 mol/L acetic acid (19.82 g/L) were used. Stock solution for calibration curve: for this, 2.4 mmol/L (0.180 g/L) of glycine was dissolved in 1 l of the extraction solution and stepwise diluted to 1.2, 0.6, 0.3, and 0.15 mmol/L.

Then, 0.5–1 g of cocoa bean protein powder sample (see Section 2.2.6) was mixed with exactly 50 mL of the extraction solution. Once all samples were covered with solution, the tubes were tightly sealed and extracted in a preheated ultrasound bath (50 °C, 60 min). After extraction, the samples were centrifuged (4000 rpm, 10 min) and the supernatant was withdrawn and directly filtered (Millipore AAWP02500, 0.8 µm)**.**

The spectrophotometric measurement was conducted at 340 nm wavelength in a spectrophotometer (Thermo Fisher Scientific, type: G10S UV-Vis). Each 100 μL of the sample/calibration solution was pipetted into two cuvettes (1 for sample and blank). After adding OPA reagent to one cuvette and the reagent for the blank to the other, the blank was measured first and then the sample. Absorbance was noted after 10 min.

For the calibration curve, 2.4 mmol/L (0.180 g/L) of glycine was dissolved in one liter of the extraction solution and gradually diluted to 1.2, 0.6, 0.3, and 0.15 mmol/L. By measuring the various dilutions, including the blank sample without glycine addition, the calibration curve was obtained. Equation (1) was applied (A content of primary amino groups expressed as glycine on a dry matter basis of the samples; VF: dilution factor applied before measuring absorbance; $x_{p}$: calculated concentration in the measured sample (mmol/L); $M_{Glycin}$: molar mass of glycine (kg/mol) (0.075 kg/mol); $V_{ex}$: amount of extraction solution used (l); $E_{P}$: mass of the sample (kg)).

(1) $$A=\frac{\left(VF\times x_{p}\right)\times V_{ex}\times M_{Glycin}}{E_{P}}$$

#### 2.2.9. Sensory Evaluation of Cocoa Liquor

A modified version of the Quantitative Descriptive Analysis according to Stone and Sidel [52] was used to evaluate the different cocoa liquor samples. A trained panel of eight judges (Cocoa and Chocolate panel of ZHAW, Zurich University of Applied Sciences) evaluated eight cocoa liquor samples (mixed samples from the three independent runs per location A and E, Figure 1, prepared according to Streule et al. [6]) from the current study (all samples underwent a quality control process to guarantee adherent food safety standards). The judges were informed about the research’s objectives prior to the study and recruited on a voluntary basis. The panel was already experienced in sensory analysis of cocoa liquor. Nevertheless, 10 training sessions were conducted prior to the test evaluation to familiarize the judges with the test samples and to train them specifically on the different intensity range of each selected criteria. The panel evaluated 12 attributes for each sample which were rated on a 10 cm continuous line scale from “0 = not perceivable” to “10 = very intense” (Table 1). The samples were presented in a sequential monadic presentation design and randomized according to a balanced design (Wiliams Latin Square). All samples were evaluated in triplicate under controlled conditions in the sensory laboratory of ZHAW. All sensory booths were equipped with tablets to enable direct data collection. The data were recorded using the sensory software Fizz Biosystems Version 2.5.1. Prior to sensory evaluation, samples were portioned and stored in a climate-controlled cabinet at 15 °C. All samples were labelled with a three-digit random code before tasting. Samples were liquefied in a heating cabinet and served for tasting at 45 °C. To avoid a carry-over effect, warm water (40 °C) was used as palate cleanser.

### 2.3. Statistics

All fermentation variations were performed three times. For normally distributed data (checked using Shapiro–Wilk test and/or Q–Q plot), a one-way ANOVA, followed by post hoc (Tukey HSD test), was applied per location. For pairwise comparisons between with/without pod storage or high/low temperatures, a t-test with Bonferroni correction was applied. Principle Component Analysis (PCA) was applied for results from process and quality parameters, targeting noticeable differences between samples. Statistical analyses were performed using RStudio (version 2023.03.0).

Regarding sensory analysis, for each attribute, the mean value was calculated from the intensity rating of the individual judges. A correlation analysis (Person (n-1)) was conducted for all sensory attributes. Based on a two-factor analysis of variance (Two-Way ANOVA/mixed model with random assessors) and by means of a post hoc test (Fisher’s L.S.D. for multiple comparisons), the significant differences between the individual test samples and attributes were evaluated. Additionally, Principal Component Analysis (PCA) and Cluster Analysis (Hierarchical Clustering) were carried out. The software program Excel (Microsoft Office Excel 365 ProPlus), the statistical software SENPAQ v.6.0 (Qi Statistics Ltd., Kent, UK), XLSTAT 2022 (Addinsof, Paris, France), and the sensory software Fizz v.2.6.1 (Biosystems) were used to analyze the panel performance and the sensory properties of the test samples.

All the tests were performed at a significance level of *p* < 0.05.

## 3. Results

### 3.1. Pod and Bean Color, Pod Weight, and Pulp Content

**Figure 5:**
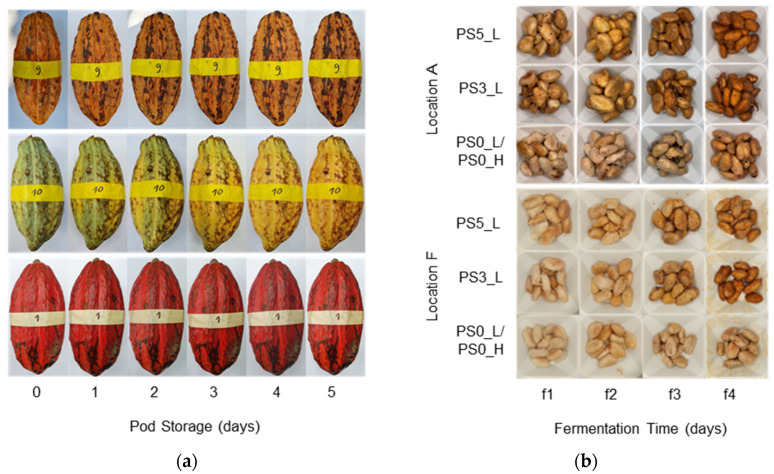
(**a**) Exemplary development of color of three pods during storage at location A; (**b**) cocoa beans from variations PS5_L, PS3_L, PS0_L/PS0_H daily during fermentation at locations A and F. PS5, PS3, and PS0 = 5, 3, and 0 days of pod storage, L and H = 60 °C and 80 °C drying temperature.

During pod storage, the cacao pods slightly changed their appearance. Yellow and red pods developed black spots during PS and green pods changed color to yellow after 5 days of PS (Figure 5a). Beans with PS turned from beige to brownish 1–2 days faster than without PS (Figure 5b).

**Figure 6:**
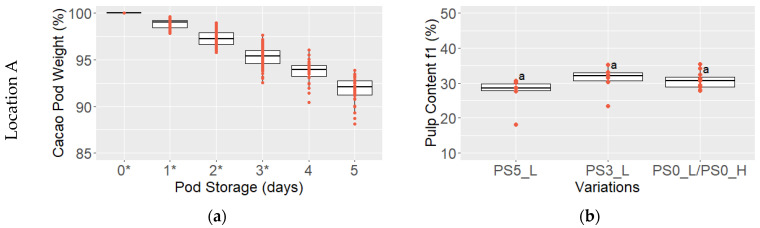

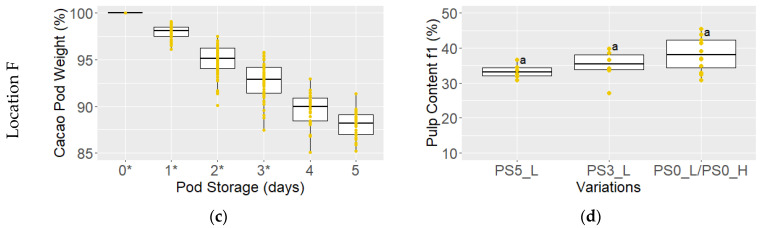
Cacao pod weight (%) during pod storage (PS) at locations A in red (**a**) and F in yellow (**c**) of variations with 3–5 days pod storage (* *n* = 120, *n* = 60) and pulp content (%) at the first day of fermentation f1 from variations PS5_L, PS3_L, PS0_L/PS0_H at locations A in red (**b**) and F in yellow (**d**) (*n* = 6 for PS5_L, PS3_L and *n* = 12 for PS0_L/PS0_H); pulp contents that do not share the same letter differ significantly (*p* < 0.05).

The weight of cacao pods decreased significantly during storage, dropping from 100% to 94.6 ± 5.5% at location A and to 92.7 ± 1.8% at location F after three days and further to 91.8 ± 1.4% at A and 87.5 ± 2.1% at F after five days (*p* < 0.05) (Figure 6a,c).

The pulp content measured on day 1 of fermentation (f1) indicated a tendency (*p* > 0.05) towards reduced pulp content with longer PS periods among all variations. Namely, variation with 5 days PS led to a slightly reduced pulp content of 27.3 ± 4.7% at location A and 33.3 ± 2.0% at location F compared to the variation without PS, resulting in values of 30.7 ± 2.4% at A and 38.3 ± 4.9% at F, respectively (Figure 6b,d). At location F, the 3 days PS also led to a rather lower pulp content (35.0 ± 4.6%) than without PS.

### 3.2. Yeast and Lactic Acid Bacteria Counts

**Figure 7:**
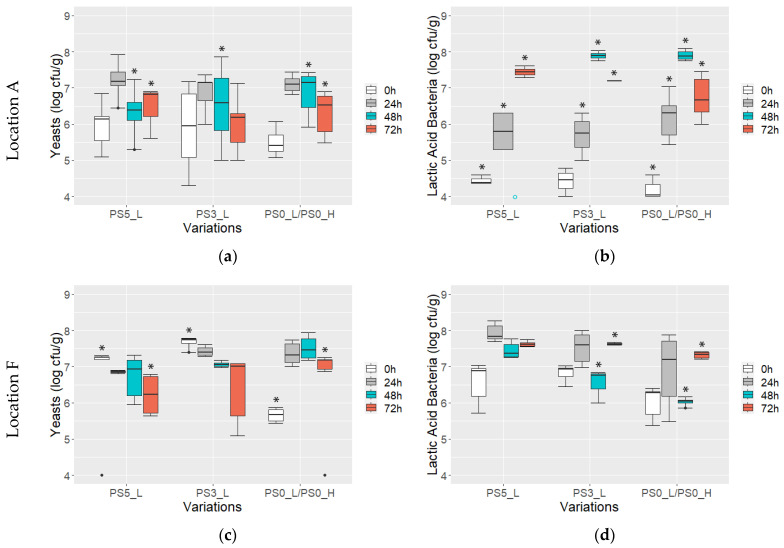
Cell counts (log cfu/g) of yeasts (**a**,**c**) and lactic acid bacteria (**b**,**d**) at location A (**a**,**b**) and F (**c**,**d**) from variations with pod storage (PS) of 5, 3, and 0 days (PS5_L, PS3_L, PS0_L/PS0_H) recorded after 0, 24, 48, and 72 h of fermentation. Sample sizes were *n* = 6 for PS5_L and PS3_L and *n* = 12 for PS0_L/PS0_H, except for cases marked with * above boxplots where 1–6 values and marked with ° all values were under detection limit.

The development of yeasts and lactic acid bacteria (LAB) during fermentation varied between locations A and F, as well as between variations fermented with pod-stored beans versus those without pod storage (Figure 7). At location A, initial cell counts were significantly lower compared to location F (yeasts, *p* = 0.017; LAB, *p* < 2 × 10^−16^). Moreover, differences were observed at both locations depending on whether the fermentation started with beans that were pod-stored or not. Particularly at location F, yeast and LAB counts were significantly higher in samples with PS (yeasts: 7.1 ± 1.2 log cfu/g, LAB: 6.7 ± 0.4 log cfu/g) than those without (yeasts: 5.7 ± 0.2 log cfu/g, LAB: 6.0 ± 0.4 log cfu/g) (*p* = 0.0042 and *p* = 0.00082, respectively).

At location A, yeast counts (Figure 7a) increased within the first 24 h and then decreased to 6.2 ± 0.7 log cfu/g with no significant difference between variations. Conversely, at location F (Figure 7c), yeast counts steadily decreased in variations with PS, with a peak observed within the first 24 h before declining. Notably, significantly lower counts were observed after 48 h of fermentation in samples with PS, with counts of 6.9 ± 0.5 log cfu/g and 7.5 ± 0.3 log cfu/g for samples with and without PS, respectively (*p* = 0.00064). End yeast counts of 6.5 ± 0.9 log cfu/g were comparable to location A.

Regarding further development of LAB, the counts rose in all variations during the first 24 h. At location A (Figure 7b), the counts continued rising until reaching a peak of 7.9 ± 0.1 log cfu/g after 48 h, except for the PS5_L variation where all values were below the detection limit. End LAB counts were slightly higher in variations with PS (7.4 ± 0.2 log cfu/g) compared to those without (6.7 ± 0.6 log cfu/g), but this difference was not significant (*p* = 0.13). At location F (Figure 7d), the LAB counts decreased after 48 h to significantly lower values than at location A (*p* = 0.0015), with significant differences also observed between variations with and without PS (*p* = 0.0021). In the last 24 h of fermentation, the LAB counts slightly increased again at location F to a similar range as at location A, with higher counts in samples with PS (7.6 ± 0.1 log cfu/g) compared to those without (7.3 ± 0.1 log cfu/g) (*p* = 0.0026).

### 3.3. Temperature of the Cocoa Bean Mass

**Figure 8:**
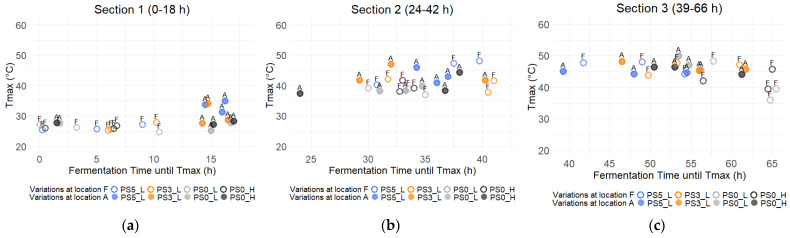
Maximal temperature Tmax (°C) during different sections of the fermentation and time until reaching Tmax (h) of variations with pod storage (PS) of 5, 3, and 0 days (PS5_L (blue), PS3_L (orange), PS0_L (grey), and PS0_H (dark grey) at location A (dots) and F (circles)). (**a**) Tmax in Section 1 from 0 to 18 h of fermentation; (**b**) Tmax in Section 2 from 24 to 42 h of fermentation; (**c**) Tmax in Section 3 from 39 to 66 h of fermentation.

Overall, no significant differences (*p* > 0.05) could be detected in the maximal reached temperatures Tmax (*y*-axis) during fermentation (45.2 ± 31 °C), nor in the time until reaching these temperatures (*x*-axis), independent of the type of variation (Figure 8). Consequently, the temperatures during fermentation were divided into three sections for more in-depth analysis.

The first section spanned from 0 to 18 h (Figure 8a), while the second section was defined as the interval from 24 to 42 h (Figure 8b). The gap between Section 1 and Section 2 was the approximate pre-drying period. Section 3 covered the time from 39 to 66 h (Figure 8c), starting with the sampling and turning in the morning. The timing of these activities (sampling and turning) varied across runs and locations, resulting in an overlap between Section 2 and Section 3. Section 3 lasted until the end of fermentation.

In the initial section, no or only slightly increased temperatures were measured since the fermentation started, independent of the variation. In only four samples with PS at location A the temperature increased slightly after 15–17 h to 31.3–35.0 °C.

Moving to the second part, the temperatures increased across all fermentations. Notably, fermentations with PS consistently reached significantly higher temperatures (*p* = 0.0018), with Tmax reaching 43.1 ± 3.2 °C (PS5_L and PS3_L), in contrast to those without PS, which reached 39.2 ± 2.0 °C.

During the final hours of fermentation, similar temperatures (45.1 ± 3.2 °C, *p* = 0.18) were reached among samples from all variations, but samples without PS required significantly more time (51–66 h) to reach Tmax than those with PS (39–62 h) (*p* = 0.0081).

### 3.4. Development of pH of Pulp and Cotyledon

The initial pulp pH was 3.8 ± 0.1, while the cotyledon pH was 6.4 ± 0.2, without differences in the variations and locations. Over the first 24 h of fermentation, the pulp pH exhibited a decrease, with the most pronounced change observed in variation PS5_L, although it did not reach statistical significance (*p* > 0.05). Subsequently, the pulp pH constantly increased again, with PS5_L having rather lower pH pulp values, reaching a final pH of 3.8 ± 0.2 for PS5_L and 4.0 ± 0.3 for PS3_L and 4.0 ± 0.2 PS0_L/PS0_H for both locations by the end of fermentation (Table 2).

In contrast, the cotyledon pH consistently decreased throughout fermentation, reaching final values of 5.1 ± 0.4 at location A and 5.6 ± 0.4 at F (*p* = 0.0047), without significant difference in the variations at 72 h of fermentation (*p* > 0.05) (Table 2). Variations with PS (PS5_L/PS3_L) at location A showed a lower pH after 24 h (*p* = 0.042) and 48 h (*p* > 0.05) of fermentation than without PS (PS0_L/PS0_H) and at location F only variation PS5_L showed rather lower pH after 48 h, but not significantly (*p* > 0.05). Upon drying, the final pH values varied slightly, with variations with PS recording 5.4 ± 0.2, while variations without PS had a pH of 5.2 ± 0.3 (*p* = 0.053) (Table 2).

### 3.5. Sugars and Organic Acids in Dried Beans

In dried beans, slight tendencies in the levels of sugars and organic acids could be observed (Table 3).

In terms of sugars, a general trend suggested that sample PS0_H exhibited slightly higher sucrose levels and slightly lower fructose and glucose levels, although these variances were not significant. However, sucrose content was significantly higher (*p* = 0.018) at location F (3.6 ± 0.6 mg/g) than at A (2.8 ± 1.0 mg/g), with PS0_H samples showing a slightly higher level at both locations, albeit not reaching significance (*p* < 0.05). The fructose content remained similar at 1.6 ± 0.4 mg/g overall, but at location F, it was significantly lower in PS0_H compared to PS5_L. The glucose content was significantly lower at location F (0.7 ± 0.3 mg/g) than at location A (1.1 ± 0.5 mg/g) (*p* = 0.019). At location A, PS0_H displayed significantly lower glucose content compared to PS3_L (*p* < 0.05).

The citric acid content was notably lower at location A compared to location F (2.6 ± 0.6 mg/g vs. 3.4 ± 0.5 mg/g, respectively; *p* = 0.0014), with no differences observed among the variations. When considering lactic acid at location A, sample PS0_H displayed a slightly lower content of 3.1 ± 1.6 mg/g compared to other variations, ranging from 4.1 to 7.8 mg/g, although this difference was only significant when compared to sample PS5_L (*p* < 0.05). At location F, the lactic acid content remained in the same range across all variations. Acetic acid levels were similar across all variations at both locations (3.9 ± 1.8 mg/g), with a minor tendency towards slightly lower acetic acid content observed in sample PS5_L at location A.

### 3.6. Protein Profile of Fermenting and Dried Beans

**Figure 9:**
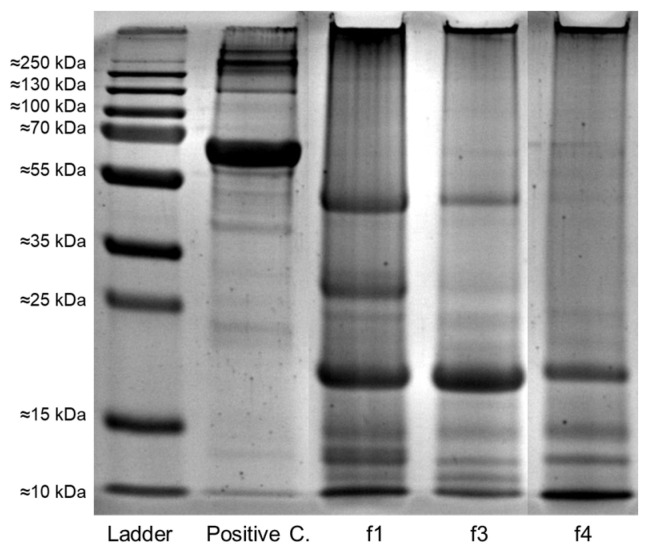
Example for SDS-PAGE (PageBlueTM Protein Staining Solution, stained gel) analyses with ladder (Dual Xtra Prestained Protein Standard (marker)), positive control and samples from fermentations days f1 (0 h), f3 (48 h), and f4 (72 h). The positive control has a molecular weight of 66.5 kDa and was used to verify the accuracy of the SDS-Page procedure.

In an SDS-PAGE, at fermentation day 1 (f1), protein bands between 15 and 25, 25 and 35, and 35 and 55 kDa were most pronounced. The longer the fermentation continued, the less intense the bands tended to appear (example in Figure 9). This mainly concerned the band between 25 and 35 kDa. The band between 35 and 55 kDa also became less visible but was often still visible at f3. Additionally, it was evident that most cocoa bean samples, regardless of location, variation, and time of measurement, exhibited three bands between 10 and 15 kDa, but in 14 samples, only two bands were discovered. From those fourteen samples, nine were from the end of drying (dEnd; 6 at location A and 6 at F), four from f4 (all at location A), and one from f1 (location A). Further, two samples exhibited four bands in the range of 10 and 15 kDa.

### 3.7. Content of Total Free Amino Acids

**Figure 10:**
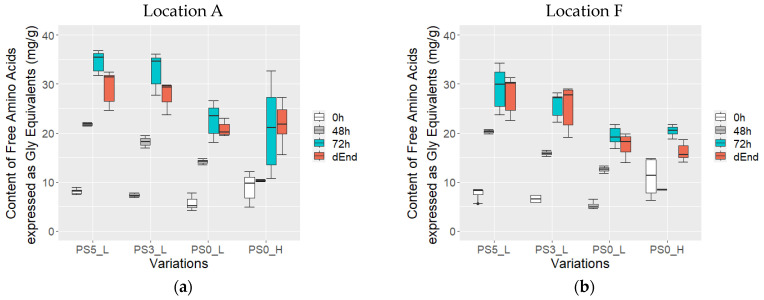
Content of total free amino acids expressed as glycine (Gly) equivalents (mg/g) in cotyledon of variations with pod storage (PS) of 5, 3, and 0 days: PS5_L, PS3_L, PS0_L, PS0_H at location A (**a**) and F (**b**) after 0 h (*n* = 6 at location A and *n* = 4 at location F), 48 h (*n* = 2), 72 h of fermentation and in beans, dried at 60 °C (L) and 80 °C (H) (dEnd, *n* = 6).

During the fermentation process, the concentration of total free amino acids exhibited a steady increase from 0 t o 72 h across all variations (Figure 10). At the fermentation start (0 h), the total free amino acid content ranged around 7.5 ± 2.4 mg/g, irrespective of the variations. Noteworthy differences emerged particularly beyond 48 h, becoming more pronounced after 72 h at both locations.

In variations where PS was performed, the total free amino acid content displayed substantial augmentation, reaching 19.0 ± 2.5 mg/g after 48 h and 30.6 ± 4.7 mg after 72 h. This was significantly higher compared to variations without PS, which recorded values of 11.4 ± 2.4 mg/g after 48 h and 20.9 ± 4.8 mg/g after 72 h (*p* < 0.05).

In dried beans, a similar trend persisted, with variations with PS exhibiting a significantly elevated total free amino acid content of 27.7 ± 3.8 mg/g compared to 19.1 ± 3.5 mg/g in variations without PS (*p* < 0.05). Notably, no significances were noted between samples PS0_L and PS0_H regarding drying temperatures of 60 and 80 °C, respectively (*p* > 0.05).

Regarding locations, notable differences were observed, particularly with higher values recorded at location A for certain variations and times. Variations PS3_L at 0 h, PS3_L and PS5_L at 72 h, and PS0_L/PS0_H in dried beans exhibited significantly higher levels than those at location F (*p* < 0.05).

### 3.8. Fermentation Index and Cut-Test

The average fermentation index (FI) of all samples was 0.7 ± 0.1. No significant differences (*p* > 0.05) in FI were detected, nor in variations, locations, or runs (Table 4).

The results of the cut-test (Table 4) revealed significant variations associated with pod storage (PS5_L, PS3_L). Samples with PS with 52.3 ± 22.6% were significantly more well-fermented (*p* = 0.0074) and with 34.9 ± 16.2% had significantly less slightly fermented beans (*p* = 0.0014) compared to those without PS (PS0_L) (22.5 ± 9.3% and 62.7 ± 9.2%, respectively). Additionally, regarding drying temperature, there was a significant increase in well-fermented beans observed at lower temperatures compared to higher temperatures (*p* = 0.0055).

However, it is noteworthy that the significant differences observed due to PS were primarily observed at location A. There, the samples with PS exhibited significantly lower shares of slightly fermented beans (25.3 ± 14.1%) compared to those without PS (67.0 ± 10.5%) (*p* = 0.0029) and significantly higher shares of well-fermented (59.7 ± 19.2%) compared to the absence of PS (20.3 ± 4.7%) (*p* = 0.012). At location F, a similar trend was observed, but without statistical significance.

Conversely, at location F, significantly more well-fermented (24.7 ± 13.4%) and fewer slightly fermented beans (58.3 ± 6.7%) were observed in samples dried at lower temperatures compared to those dried at higher temperatures (1.7 ± 2.1% and 93.3 ± 3.2%, respectively) (*p* = 0.043 and *p* = 0.0012, respectively). At location A, there were also tendencies indicating a higher share of well-fermented beans in samples dried at lower temperatures (60 °C instead of 80 °C), although without significance.

Violet beans were consistently present at around 14.3 ± 13.4% in all variations and both locations. No slaty or moldy beans were detected in either sample.

### 3.9. Sensory Evaluation of Cocoa Liquor

With the exception of the floral aroma attribute, all selected descriptors contributed significantly to product differentiation (*p* < 0.05) (Table 5). Regarding taste and trigeminal sensation, samples with a high drying temperature (80 °C) from both locations showed the most intense bitterness and astringency, whereas the most intense acidity was observed in the cocoa sample from location A without PS and low drying temperature (60 °C). Fruity notes tended to be less intense at the higher drying temperature. Samples from location F exhibited a tendency towards more intense nutty notes compared to samples from location A.

Strong correlation (*r* > 0.7) could be observed between the attributes astringency and bitterness, between astringency and green/herbal, and between acidity and fruity. Overall, only a few strong correlations were detectable across all attributes, which means that each attribute makes an important contribution to product differentiation (Table S1).

### 3.10. Principal Component Analysis

#### 3.10.1. Focus on Pod Storage

**Figure 11:**
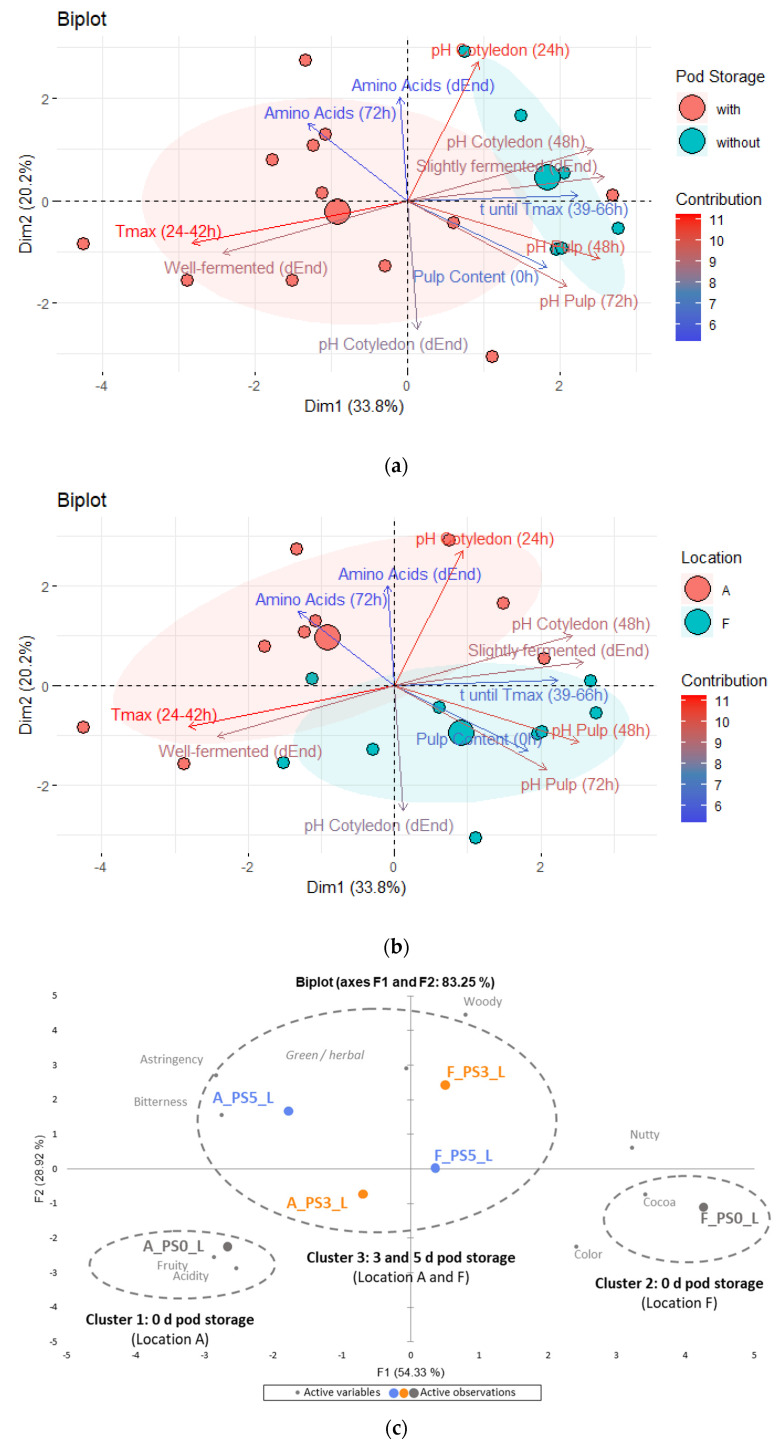
Principal Component Analysis focusing on variations PS5_L, PS3_L, PS0_L with variables during fermentation and of dried beans (pulp content (0 h), maximal temperature Tmax (24–42 h), time t until Tmax (39–66 h), pH pulp at 48 h and 72 h, pH cotyledon at 24 h, 48 h and dEnd, well-fermented and slightly fermented beans (dEnd), total free amino acids at 72 h and dEnd) grouped by (**a**) with pod storage (PS) of 5 and 3 days in red, and without pod storage (PS of 0 days) in blue (**b**), with location A (red) and F (blue); (**c**) Principal Component Analysis focusing on sensory characteristics from samples PS5_L (blue), PS3_L (orange), PS0_L (grey) at locations A and F. L = drying at 60 °C, H = drying at 80 °C. The variable ‘green/herbal’ is shown in italics as it is not well represented in the first two dimensions, F1 and F2.

Biplots were created with parameters during fermentation (pulp content, maximal temperature in Section 2, time until maximal temperature in Section 3, pH in pulp after 48 h and 72 h, pH in cotyledon after 24 h and 48 h, amino groups after 72 h) and after drying (pH in cotyledon, slightly and well-fermented beans, total free amino acids) which showed significant differences or tendency of differences between the three samples with different PS times (PS5_L, PS3_L, PS0_L). The first dimension explained 33.8% of the variance. The distinction between the two groups, one with PS (PS3_L, PS5_L) and one without (PS0_L), was clearly evident (Figure 11a). Variations with PS were especially characterized by higher temperature during fermentation (Section 2) and more well-fermented beans in the cut-test. Additionally, variations in locations were also observable (Figure 11b).

The PCA (Figure 11c) was plotted with the significantly differing sensory attributes from the samples with different PS times and same drying temperature (60 °C), namely PS5_L, PS3_L, PS0_L. The first dimension, which accounted for 54.33% of the variance, depicted “cocoa” and “nutty” attributes on one side (left) and “bitterness” and “astringency” on the opposite side (right). Cluster analysis (Figure S1) further explained the groups signed in Figure 11c, indicating that samples lacking PS (PS0_L from locations A and F) markedly contrasted with those having 3 and 5 days of PS (A_PS5_L, A_PS3_L from location A and F_PS5_L, F_PS3_L from location F). Moreover, the biplot illustrated that samples from different locations exhibited the most pronounced disparities when no PS was carried out. Specifically, sample A_PS0_L was strongly characterized by intense acidity and fruity notes, while sample F_PS0_L was predominantly characterized by intense cocoa and nutty flavors.

#### 3.10.2. Focus on Drying Temperatures

**Figure 12:**
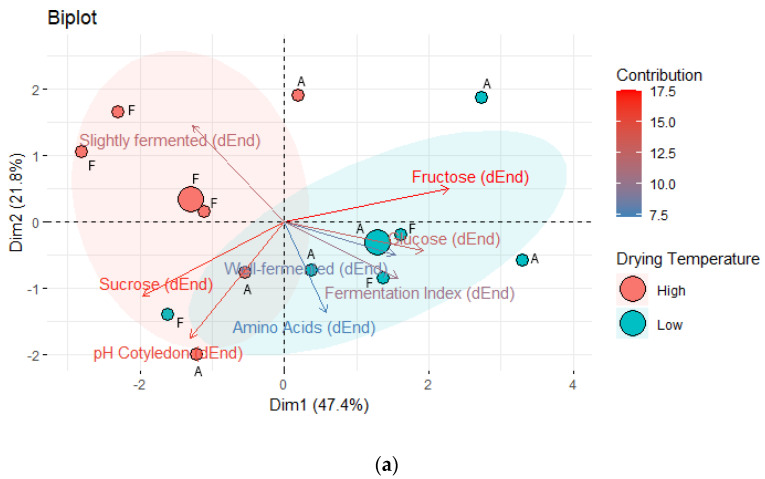

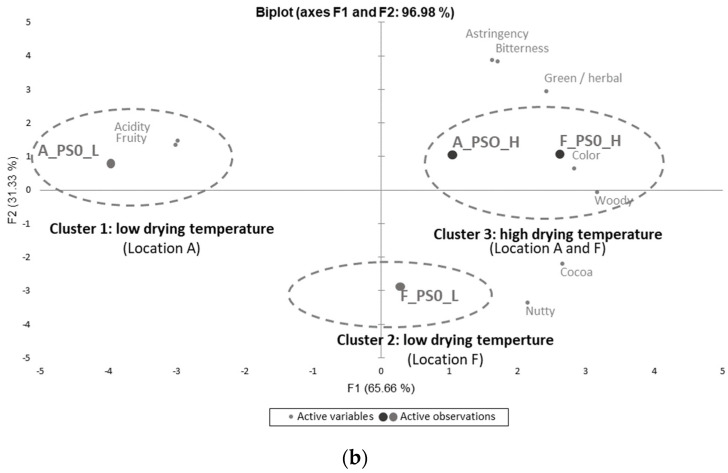
(**a**) Principal Component Analysis focusing on variations PS0_L (red), PS0_H (blue) with variables from dried beans (pH cotyledon, well-fermented and slightly fermented beans, fermentation index, total free amino acids, sucrose, fructose, glucose) grouped by drying temperature of both locations A and F; (**b**), Principal Component Analysis focusing on sensory characteristics from samples PS0_L (light grey), PS0_H (dark grey) at locations A and F. L = drying at 60 °C, H = drying at 80 °C.

A biplot was created with parameters after drying (pH in cotyledon, slightly and well-fermented beans, fermentation index, total free amino acids, sucrose, fructose and glucose content) which showed significant differences or tendency of differences between the samples without PS and dried at two different temperatures, 60 and 80 °C (PS0_L, PS0_H) (Figure 12a). The first dimension explained 47.4% of the variance. Both variations can be divided into two groups. While the samples with high drying temperature were characterized by rather more slightly fermented beans, more sucrose, and a higher pH value in cotyledon, regarding location, no clear difference was visible.

Also in the sensory results, the samples could have been divided into groups (Figure 12b, Cluster analysis in Figure S2). Cluster 3 represented samples with high drying temperature of locations A and F (A_PS0_H and F_PS0_H). These samples were characterized by a dark color, intense bitterness, and astringency. Cluster 1 represented a sample from location A with low drying temperature, characterized by a strong acidity and fruitiness, whereas Cluster 2 was represented by a sample with low drying temperature form location F and characterized by intense cocoa and nutty notes.

## 4. Discussion

### 4.1. Influence of Pod Storage on Fermentation and End Quality of Cocoa Beans

The color of the cacao pods underwent changes during storage, accompanied by the detection of dark spots on some pods. These alterations can be attributed to a normal biological decomposition of the cells, as well as the activity of microorganisms attacking the pods, leading to a deterioration and discoloration of the pods. This has also been described and observed by Hinneh et al. [33]. During fermentation, pod-stored beans changed in color faster compared to the beans without pod storage (PS). This accelerated change may stem from biochemical transformations (e.g., cellular degradation, inversion of sucrose) occurring within pods and pulp, a hypothesis supported by previous research [31,33]. In this study, only a decline in the pod weight was observed, indicating a transformation in the fruit, such as water loss. Further transformations during storage of the cocoa pods (e.g., internal atmosphere [53]) should be investigated in detail in future studies. As for pulp content, only a slight reduction in pod-stored beans was observed, albeit not statistically significant. It is important to note that our sample size was limited to just 10 beans and should be expanded in future research to enhance the robustness of these results. These findings align with those of Hinneh et al. [33], who observed more dry beans with a PS of 3 days and Biehl et al. [31], who documented a reduced dry matter in pod-stored beans.

The cell counts were higher at the beginning of the fermentation, particularly in samples with PS at location F, indicating that microorganisms accumulated on the pod surface during storage are likely to have come into contact with the pulp and be working as starters for the fermentation. This observation aligns with previous studies indicating cacao pods as the primary source of inoculum for spontaneous fermentation [10].

Additionally, it suggests that PS may indeed influence the fermentation process. However, despite these initial observations, only minor differences were detected in the parameters monitored throughout the fermentation process and in the quality of dried beans between fermentations with and without pod-stored beans.

Fermentation could potentially be accelerated by removing part of the cocoa pulp [29] and there were indications that PS before fermentation could also influence the process. The observations showed a faster increase in temperature and decrease in pH in the cotyledon, particularly noticeable in fermentations with pod-stored beans [54]. The rather lower pulp content at the beginning of the fermentation may suppress the anaerobic phase of fermentation [37] and lead to a faster temperature increase. In this study, an initial increase in temperature was observed, especially evident at location A, during the first part of fermentation with pod-stored beans. Subsequently, in the second part of fermentation, higher maximal temperatures were reached with pod-stored beans. Other studies also reported a faster temperature increase in fermentations with pod-stored beans (4 and 6 days PS) within the first 24 h [54]. This might be due to a rather lower pulp content, resulting in a better aeration and reduced viscosity in the fermenting mass of pod-stored beans [54]. This condition could create a more favorable aerobic environment for microorganisms, particularly acetic acid bacteria, which are strictly aerobic. These bacteria oxidize ethanol into acetic acid, resulting in an exothermic reaction and temperature increase and production of acids, which diffuse into the cotyledon and reduce pH [29]. Although acetic acid bacteria were not monitored in this current study, a noteworthy correlation was found between maximum temperatures and lower pH values in cotyledon after 48 h (*r* = −0.66, *p* < 0.05), providing further evidence of slightly accelerated fermentation. Additionally, Bariah et al. [54] observed a faster decrease in cotyledon pH, particularly after 24 h and 48 h of fermentation with pod-stored beans. PS only influenced the pH in dried beans slightly, and no significant differences in organic acids were detected in samples with versus without pod-stored beans. Therefore, a reduced acidity with PS (one method of pulp pre-conditioning) was not clearly reached, as mentioned in previous studies [30,31].

PS further may influence free fatty acids and protein contents [34]. In this study, proteins seemed to be degraded, as interpreted by disappearing bands in SDS-Page analysis from samples taken during fermentation. Especially during ongoing fermentations, disappearing of bands between 25 and 35 kDa started after 48 h, which might be assigned to the polypeptide subunit of vicilin-like globulin with a molecular weight of 31 kDa [55]. Further, bands with 47 kDa, that may be attributed to the same polypeptides, seemed to be hydrolyzed in some samples after 72 h of fermentation, probably due to proteolytic activities and shown by disappearing bands on the SDS-Page. In further studies, proteins of 21 kDa (albumin) started disappearing only after 4 days of fermentation [55]. Similar protein profiles were found at both locations regardless the variations. Also, Hue et al. [55] observed similar profiles in cocoa—no matter what origin and phenotype. In this current study, the bands were similarly not visible per repetitions and variations; also, no differences between variations were visible. This might be due to the assumption of Kumari et al. [56] that proteolytic activities might occur differently and possibly sequential during fermentation. These activities are crucial for the development of the typical cocoa flavor, as free amino acids serve as primary precursors for the Maillard reactions [57].

Even though no clear differences in protein profiles were visible, higher amino acids in samples with PS were measured in this study. This was also observed by Afoakwa et al. [34], who attributed it to the initiation of protease activity within the pods, thereby starting the proteolysis process and consequently leading to the release of peptides and free amino acids. In Hinneh et al. [33], it is also significant that more free amino acids were observed with 7 days of PS and might have had therefore also a positive effect on the formation of volatiles. Nevertheless, the total free amino acid content underwent significant changes throughout fermentation, experiencing a notable increase attributed to the protease activity during the fermentation process [15]. Further, slight differences in locations in free amino acid content were detected, which is in line with previous observations [55] that geographical origins influence amino acid contents.

PS can lead to an increase in the formation of volatiles due to elevated levels of free amino acids and can influence the dynamics of sugar degradation [33]. However, in this study, no clear differences in sugars among dried beans with and without PS were found. Moreover, Afoakwa et al. [34] suggested that PS does not significantly impact sugar content; instead, it was assumed that fermentation primarily influences the changes in sugar composition. Although samples subjected to PS have been noted for their prevalence of, e.g., esters (fruity/floral/sweet) [58], this current investigation did not reveal clear differences in fruity and floral attributes between samples with and without PS. Nonetheless, a noticeable impact was evident across all sensory data. Samples without PS from both locations strongly differed from the samples with PS, likewise from both locations. In future research, the evaluation of volatile compounds would be advantageous for a more profound understanding of the influence of PS on specific sensory attributes.

Samples subjected to PS exhibited significantly more well-fermented and less slightly fermented beans in the cut-test compared to those without PS. This trend aligns with findings from prior research, which also noted higher proportions of well-fermented or brown beans after 3, 7, and 14 days of PS [20,59]. Additionally, an absence of moldy beans in all samples was observed, in contrast to prior findings [33,37], where increased amounts of moldy beans with longer PS durations were reported. This disparity could potentially be attributed to the use of pods in optimal physical condition without damage, such as cuts from machetes during harvesting or diseases. The incidence of mold is known to depend on damaged pods and on damaging the beans during harvesting [60]. Generally, as the duration between pod ripeness and harvesting increases, there is a rise in pod diseases and the likelihood of bean germination [61]. The physical state and condition of the pods are crucial factors, although it is worth noting that temperature and weather conditions also influence fungal contamination [41]. Contrary to previous research [20,62], no significant differences in fermentation indexes between samples with and without PS were found. Another point to mention is that independent runs of the same process variation also differed, as evidenced by the high standard deviation in some of the results. This is an important aspect to consider for further studies due to variations in climatic conditions during each repetition, which also influences the post-harvesting process and quality [61,63].

### 4.2. Influence of Drying Temperature on End Quality of Cocoa Beans

The results demonstrated notable differences in cocoa bean quality between samples dried at higher and lower temperatures. While some variations were observed during fermentation between samples PS0_L and PS0_H, it is important to note that these could be attributed to normal variations inherent in experimental repetitions.

It is particularly noteworthy that the samples dried at a higher temperature of 80 °C exhibited a slightly higher sucrose content and lower levels of fructose and glucose, but without statistical significance. It is well known that sucrose is enzymatically converted into fructose and glucose during fermentation via invertase activity [61] and its activity presumably disappears with increasing temperature [64]. Thus, the persistence of high sucrose content could potentially signify a weaker fermentation, as noted in prior research [15,16], rather than being directly attributed to the drying temperature. Moreover, the cocoa liquor produced from the higher-temperature dried samples (PS0_H) was noted to possess a more pronounced bitter and astringent profile, which aligns with findings suggesting a weaker fermentation [16]. But also, the presence of, e.g., polyphenols and 2,5-diketopiperazines (DKP) can contribute to bitterness and astringency [65,66] Notably, DKP formation can occur even at low temperatures (<60 °C) [67]. However, it is important to note that these compounds and enzymes, like polyphenol oxidase, which plays a role in reducing bitterness and astringency and whose activity decreases with increasing temperature [68], were not included in this study. Consequently, the correlation between drying temperature, drying duration (e.g., drying at 80 °C varied between 4 and 8.5 h), and these sensory attributes remains unclear.

Additionally, the lactic acid content at location A was marginally lower for PS0_H. Furthermore, the results of the cut-test indicated a marginally higher incidence of slightly fermented beans and a slightly lower occurrence of well-fermented beans in samples subjected to lower drying temperatures. These findings suggest that the disparity in fermentation time between samples PS0_H and PS0_L may have exerted a more significant influence on the dried beans than the drying temperature itself, considering that samples (PS0_H) were dried approximately 8–10 h earlier than those subjected to lower temperatures.

In terms of acetic acid content in the dried beans, which can be influenced by drying temperature and method [16,44,69], no significant difference was observed. However, samples dried at lower temperatures were found to possess slightly higher acidity compared to those dried at higher temperatures, with significance noted at location A, consistent with findings by Streule et al. [16]. Nonetheless, uncertainties remain regarding the comparability of drying performance between samples subjected to high and low temperatures. Notably, the dryer for the PS0_H samples was only filled to one-third of its surface area, with the remaining two-thirds covered by jute bags. As highlighted in previous reviews [70], crucial factors in artificial drying not only include temperature and airflow but also the surface area of the product exposed to air and the size of bean layers. Therefore, further investigations are suggested to explore the impact of bean quantity in the dryer on overall quality.

## 5. Conclusions

In conclusion, the explorative investigation suggests that pod storage (both 3 days and 5 days) offers potential benefits in improving the quality of the end-product, particularly in terms of fermented and dried beans. The analysis of process parameters and chemical composition revealed minimal differences, but notably, pod storage demonstrated positive effects, such as improved cut-test results. Additionally, sensory evaluation indicated that the impact of location was mitigated with pod storage, suggesting that pod storage could serve as a tool to standardize quality. However, clear differences in location were observed in samples without pod storage. Conducting training sessions for farmers to visually identify undamaged, healthy, and ripe cocoa pods, coupled with guidance on optimal pod storage durations, could be an effective method for enhancing cocoa bean quality. Exploring further analytical approaches to monitor pod storage and its potential could offer valuable insights for future studies. While no adverse effects were observed with a drying temperature of 80 °C, as evidenced by the results of the cut-test and fermentation index, significant differences emerged in sensory evaluations between samples dried at 80 °C and 60 °C. This disparity could be attributed to variations in fermentation duration, with the 60 °C samples undergoing longer fermentation periods (approximately 8–10 h) due to the drying process of the 80 °C samples. However, it remains unclear whether the elevated drying temperature caused the heightened bitterness and astringency. Further investigations are suggested, including simultaneous use of two dryers with an equal quantity of beans in each and potentially increasing the sample size. In the future, a more balanced test design should be applied and consider all influencing factors, aiming to examine individual effects more precisely.

Additionally, noteworthy differences were detected within independent runs, likely influenced by weather conditions and variations in post-drying methods. Therefore, future studies should always prioritize conducting independent runs to ensure reliable results.

In summary, these findings provide valuable insights into the effects of pod storage and drying temperatures on cocoa quality, offering valuable guidance to enhance cocoa production practices.

## Figures and Tables

**Table 1:** Sensory vocabulary, definition, and scale anchor points.

Category	Attributes	Definition	Scale
Appearance	Color	Color impression on the surface of the liquefied cocoa mass	Light–dark
Texture	Viscosity	Flow behavior of the cocoa liquor on the palate	Low–high
	Finesse	Perception of the size and quantity of particles in the melted cocoa liquor	Coarse–fine
Basic taste	Acidity	Basic taste, perception of the intensity of acidity like a solution of citric acid or acetic acid	Low–intense
	Bitterness	Basic taste, perception of the intensity of bitterness like a caffein solution	Low–intense
Trigeminal sensation	Astringency	Dry and rough mouthfeel	Low–intense
Aroma (retronasal)	Floral	Aroma perception, reminiscent of dried flowers/orange blossom	Low–intense
	Fruity	Aroma perception, reminiscent of citrus fruits lemon/grapefruit/bergamot	Low–intense
	Cocoa	Aroma perception, reminiscent of cocoa powder	Low–intense
	Nutty	Aroma perception, reminiscent of unroasted walnuts	Low–intense
	Woody	Aroma perception, reminiscent of dry wood	Low–intense
	Green/herbal	Aroma perception, reminiscent of dried herbs	Low–intense

**Table 2:** Pulp pH and cotyledon pH during fermentation (0 h, 24 h, 48 h, 72 h) and of dried beans (dEnd) of variations with pod storage (PS) of 5, 3, and 0 days dried at 60 °C (L) and 80 °C (H): PS5_L (*n* = 3), PS3_L (*n* = 3), PS0_L, and PS0_H (during fermentation, *n* = 6; *n* = 3 of dried bean each) at location A and F.

Location	Variation	Pulp pH				Cotyledon pH				
		0 h	24 h	48 h	72 h	0 h	24 h	48 h	72 h	dEnd
A	PS5_L	3.9 ± 0.1 ^a^	3.3 ± 0.3 ^a^	3.4 ± 0.2 ^a^	3.6 ± 0.2 ^a^	6.5 ± 0.1 ^a^	6.3 ± 0.1 ^a^	5.5 ± 0.6 ^a^	5.1 ± 0.3 ^a^	5.2 ± 0.1 ^a^
	PS3_L	4.0 ± 0.2 ^a^	3.5 ± 0.4 ^a^	3.6 ± 0.3 ^a^	3.8 ± 0.3 ^a^	6.5 ± 0.1 ^a^	6.3 ± 0.2 ^a^	5.7 ± 0.7 ^a^	5.1 ± 0.2 ^a^	5.3 ± 0.1 ^a^
	PS0_L	3.8 ± 0.1 ^a^	3.5 ± 0.3 ^a^	3.9 ± 0.3 ^a^	4.0 ± 0.2 ^a^	6.5 ± 0.2 ^a^	6.5 ± 0.1 ^a^	6.0 ± 0.4 ^a^	5.1 ± 0.5 ^a^	5.0 ± 0.2 ^a^
	PS0_H									5.3 ± 0.4 ^a^
F	PS5_L	3.8 ± 0.2 ^a^	3.5 ± 0.3 ^a^	3.8 ± 0.1 ^a^	4.0 ± 0.1 ^a^	6.3 ± 0.1 ^a^	6.2 ± 0.1 ^a^	5.9 ± 0.5 ^a^	5.8 ± 0.6 ^a^	5.6 ± 0.3 ^a^
	PS3_L	3.8 ± 0.1 ^a^	3.6 ± 0.3 ^a^	4.0 ± 0.1 ^a^	4.3 ± 0.3 ^a^	6.3 ± 0.1 ^a^	6.3 ± 0.2 ^a^	6.0 ± 0.1 ^a^	5.5 ± 0.4 ^a^	5.5 ± 0.3 ^a^
	PS0_L	3.7 ± 0.1 ^a^	3.6 ± 0.2 ^a^	3.9 ± 0.2 ^a^	4.1 ± 0.2 ^a^	6.2 ± 0.1 ^a^	6.2 ± 0.1 ^a^	6.1 ± 0.1 ^a^	5.5 ± 0.4 ^a^	5.4 ± 0.2 ^a^
	PS0_H									5.2 ± 0.2 ^a^

Values in same column of same location with same letters do not significantly differ (*p* < 0.05).

**Table 3:** Sugars (sucrose, fructose, glucose) and organic acids (citric acid, lactic acid, acetic acid) (mg/g) in dried beans at location A and F of variations with pod storage (PS) of 5, 3, and 0 days, dried at 60 °C (L) and 80 °C (H) (PS5_L, PS3_L, PS0_L, PS0_H), *n* = 3.

Location	Variation	Sugars (mg/g)			Organic Acids (mg/g)		
		Sucrose	Fructose	Glucose	Citric Acid	Lactic Acid	Acetic Acid
A	PS5_L	2.9 ± 0.3 ^a^	1.8 ± 0.2 ^a^	1.2 ± 0.1 ^ab^	2.6 ± 0.6 ^a^	6.7 ± 1.1 ^a^	2.3 ± 1.0 ^a^
	PS3_L	2.3 ± 0.8 ^a^	2.0 ± 0.0 ^a^	1.4 ± 0.3 ^a^	2.9 ± 0.4 ^a^	6.1 ± 0.8 ^ab^	3.8 ± 1.0 ^a^
	PS0_L	2.3 ± 0.9 ^a^	1.9 ± 0.5 ^a^	1.3 ± 0.5 ^ab^	2.3 ± 0.6 ^a^	4.9 ± 0.9 ^ab^	5.5 ± 2.0 ^a^
	PS0_H	3.6 ± 1.4 ^a^	1.4 ± 0.2 ^a^	0.5 ± 0.4 ^b^	2.6 ± 0.8 ^a^	3.1 ± 1.6 ^b^	3.5 ± 0.9 ^a^
F	PS5_L	3.4 ± 0.7 ^a^	1.8 ± 0.3 ^a^	0.8 ± 0.0 ^a^	3.6 ± 0.7 ^a^	3.9 ± 1.7 ^a^	4.3 ± 2.0 ^a^
	PS3_L	3.7 ± 0.6 ^a^	1.5 ± 0.1 ^ab^	0.7 ± 0.3 ^a^	3.0 ± 0.6 ^a^	4.5 ± 0.9 ^a^	5.0 ± 2.9 ^a^
	PS0_L	3.3 ± 0.7 ^a^	1.5 ± 0.5 ^ab^	0.8 ± 0.4 ^a^	3.3 ± 0.3 ^a^	5.3 ± 0.4 ^a^	3.6 ± 2.3 ^a^
	PS0_H	4.0 ± 0.3 ^a^	1.0 ± 0.1 ^b^	0.4 ± 0.3 ^a^	3.8 ± 0.3 ^a^	4.4 ± 0.9 ^a^	3.3 ± 1.6 ^a^

Values in same column of same location with same letters do not significantly differ (*p* < 0.05).

**Table 4:** Fermentation index (*n* = 9) and share of well-fermented, slightly fermented, and violet beans (%) of cut-test (*n* = 3) of variations with pod storage (PS) of 5, 3, and 0 days and dried at 60 °C (L) and 80 °C (H): PS5_L, PS3_L, PS0_L, PS0_H at location A and F.

Location	Variation	FermentationIndex	Cut-Test		
			Well-Fermented Beans (%)	Slightly Fermented Beans (%)	Violet Beans (%)
A	PS5_L	0.8 ± 0.1 ^a^	53.0 ± 24.6 ^ab^	34.3 ± 15.3 ^bc^	12.7 ± 13.4 ^a^
	PS3_L	0.7 ± 0.0 ^a^	66.3 ± 13.6 ^a^	16.3 ± 4.7 ^c^	17.3 ± 9.5 ^a^
	PS0_L	0.8 ± 0.1 ^a^	20.3 ± 4.7 ^bc^	67.0 ± 10.5 ^a^	12.7 ± 8.1 ^a^
	PS0_H	0.8 ± 0.1 ^a^	9.7 ± 8.5 ^c^	62.3 ± 12.0 ^ab^	28.0 ± 20.3 ^a^
F	PS5_L	0.7 ± 0.1 ^a^	50.7 ± 30.7 ^a^	40.0 ± 18.1 ^b^	9.3 ± 12.9 ^a^
	PS3_L	0.7 ± 0.0 ^a^	39.0 ± 22.5 ^a^	49.0 ± 3.5 ^b^	12.0 ± 19.1 ^a^
	PS0_L	0.7 ± 0.1 ^a^	24.7 ± 13.4 ^a^	58.3 ± 6.7 ^b^	17.0 ± 17.0 ^a^
	PS0_H	0.7 ± 0.0 ^a^	1.7 ± 2.1 ^a^	93.3 ± 3.2 ^a^	5.0 ± 3.0 ^a^

Values in same column of same location with same letters do not significantly differ (*p* < 0.05).

**Table 5:** Mean values of sensory attributes, *p-*values from ANOVA, and results from Post hoc test (Fisher’s LSD) of samples from variations with pod storage (PS) of 5, 3, and 0 days, dried at 60 °C (L) or 80 °C (H) at locations A and F.

Category	Attributes	*p*-Values	Location A				Location F			
			PS5_L	PS3_L	PS0_L	PS0_H	PS5_L	PS3_L	PS0_L	PS0_H
Appearance	Color	<0.05	4.7 ^cd^	4.4 ^d^	5.3 ^c^	6.1 ^b^	5.0 ^cd^	4.8 ^cd^	6.2 ^b^	7.6 ^a^
Texture	Viscosity	<0.05	5.8 ^a^	3.5 ^d^	3.7 ^cd^	4.8 ^abcd^	4.2 ^bcd^	4.3 ^bcd^	4.8 ^abc^	5.2 ^ab^
	Finesse	<0.05	3.3 ^c^	7.2 ^a^	7.3 ^a^	6.6 ^a^	6.1 ^ab^	5.4 ^b^	7.0 ^a^	7.1 ^a^
Basic taste	Acidity	<0.05	4.2 ^bcd^	4.7 ^bc^	6.2 ^a^	4.0 ^bcd^	4.8 ^b^	3.9 ^cd^	3.6 ^d^	3.5 ^d^
	Bitterness	<0.05	5.0 ^abc^	4.4 ^c^	4.8 ^bc^	5.6 ^ab^	4.5 ^c^	4.6 ^c^	4.3 ^c^	5.6 ^a^
Trigeminal sensation	Astringency	<0.05	5.2 ^abc^	4.8 ^cd^	4.9 ^cd^	5.8 ^a^	4.8 ^cd^	5.0 ^bcd^	4.3 ^d^	5.8 ^ab^
Aroma (retronasal)	Floral	0.12	3.4 ^a^	2.6 ^a^	2.2 ^a^	2.4 ^a^	3.0 ^a^	2.2 ^a^	2.3 ^a^	2.5 ^a^
	Fruity	<0.05	2.7 ^bc^	3.2 ^b^	4.7 ^a^	2.1 ^cd^	2.5 ^bc^	2.1 ^cd^	1.6 ^d^	1.3 ^d^
	Cocoa	<0.05	4.7 ^d^	4.9 ^cd^	4.6 ^d^	5.6 ^ab^	5.1 ^bcd^	5.0 ^cd^	5.8 ^a^	5.5 ^abc^
	Nutty	<0.05	3.2 ^c^	3.3 ^c^	3.2 ^c^	3.9 ^bc^	4.1 ^ab^	4.3 ^ab^	4.8 ^abc^	4.2 ^ab^
	Woody	<0.05	3.5 ^ab^	2.9 ^abc^	1.9 ^c^	3.0 ^ab^	2.7 ^bc^	3.8 ^a^	2.8 ^abc^	3.3 ^ab^
	Green/herbal	<0.05	2.2 ^c^	2.0 ^c^	2.2 ^c^	3.4 ^ab^	2.3 ^bc^	2.0 ^bc^	2.1 ^c^	3.8 ^a^

Mean values with the same letter in a row do not significantly differ (*p* < 0.05).

## Data Availability

The original contributions presented in the study are included in the article/supplementary material, further inquiries can be directed to the corresponding author.

## Supplementary Materials

The following supporting information can be downloaded at: https://www.mdpi.com/article/10.3390/foods13101536/s1, Figure S1: Results from Cluster Analysis based on sensory data of samples from variations with pod storage (PS) of 5 (blue), 3 (orange), and 0 days (grey), dried at 60 °C (L) from locations A and F; Figure S2: Results from Cluster Analysis based on sensory data of samples from variations without pod storage (PS0), dried at 60 °C (L, light grey) and at 80 °C (H, dark grey) from locations A and F; Table S1: Correlation matrix of the sensory attributes. The values in the matrix represent the Pearson correlation coefficient between the different sensory attributes. Values in bold are different from 0 with significance level alpha = 0.05.
