# Supplementary material for: Effect of Pod Storage and Drying Temperature on Fermentation Dynamics and Final Bean Quality of Cacao Nacional in Ecuador
Source: Foods. 2024 May 15;13(10):1536. doi: 10.3390/foods13101536 (PMC11121351; doi:10.3390/foods13101536)
Supplement: Supplementary file 1 [file foods-13-01536-s001.zip › foods-2986217-supplementary.pdf]

Supplementary Material

Table S1: Correlation matrix of the sensory attributes. The values in the matrix represent the Pearson correlation coefficient between the different sensory attributes. Values in bold are different from 0 with significance level alpha = 0.05.

Variables	Color	Viscosity	Finesse	Acidity	Bitterness	Astringency	Floral	Fruity	Cocoa	Nutty	Woody	Green / herbal
Color	**1.00**	0.39	0.45	-0.48	0.64	0.50	-0.34	-0.57	**0.74**	0.51	0.05	**0.82**
Viscosity	0.39	**1.00**	-0.62	-0.64	0.49	0.38	0.52	-0.60	0.29	0.19	0.59	0.40
Finesse	0.45	-0.62	**1.00**	0.18	-0.01	-0.04	**-0.75**	0.08	0.42	0.25	-0.57	0.23
Acidity	-0.48	-0.64	0.18	**1.00**	-0.26	-0.25	-0.05	**0.96**	-0.70	-0.66	**-0.81**	-0.46
Bitterness	0.64	0.49	-0.01	-0.26	**1.00**	**0.96**	0.00	-0.28	0.24	-0.11	0.23	**0.91**
Astringency	0.50	0.38	-0.04	-0.25	**0.96**	**1.00**	0.02	-0.27	0.15	-0.14	0.33	**0.89**
Floral	-0.34	0.52	**-0.75**	-0.05	0.00	0.02	**1.00**	-0.04	-0.33	-0.35	0.29	-0.20
Fruity	-0.57	-0.60	0.08	**0.96**	-0.28	-0.27	-0.04	**1.00**	**-0.79**	**-0.78**	**-0.71**	-0.53
Cocoa	**0.74**	0.29	0.42	-0.70	0.24	0.15	-0.33	**-0.79**	**1.00**	**0.81**	0.18	0.53
Nutty	0.51	0.19	0.25	-0.66	-0.11	-0.14	-0.35	**-0.78**	**0.81**	**1.00**	0.29	0.26
Woody	0.05	0.59	-0.57	**-0.81**	0.23	0.33	0.29	**-0.71**	0.18	0.29	**1.00**	0.29
Green / herbal	**0.82**	0.40	0.23	-0.46	**0.91**	**0.89**	-0.20	-0.53	0.53	0.26	0.29	**1.00**

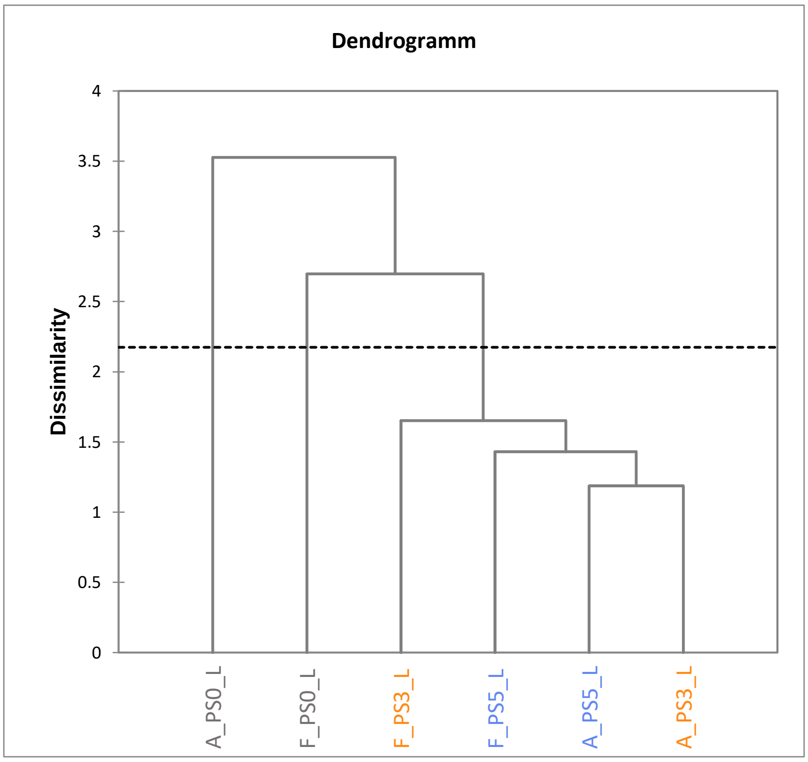

Figure S1: Results from Cluster Analysis based on sensory data of samples from variations with pod storage (PS) of 5 (blue), 3 (orange), and 0 days (grey), dried at 60 °C (L) from locations A and F.

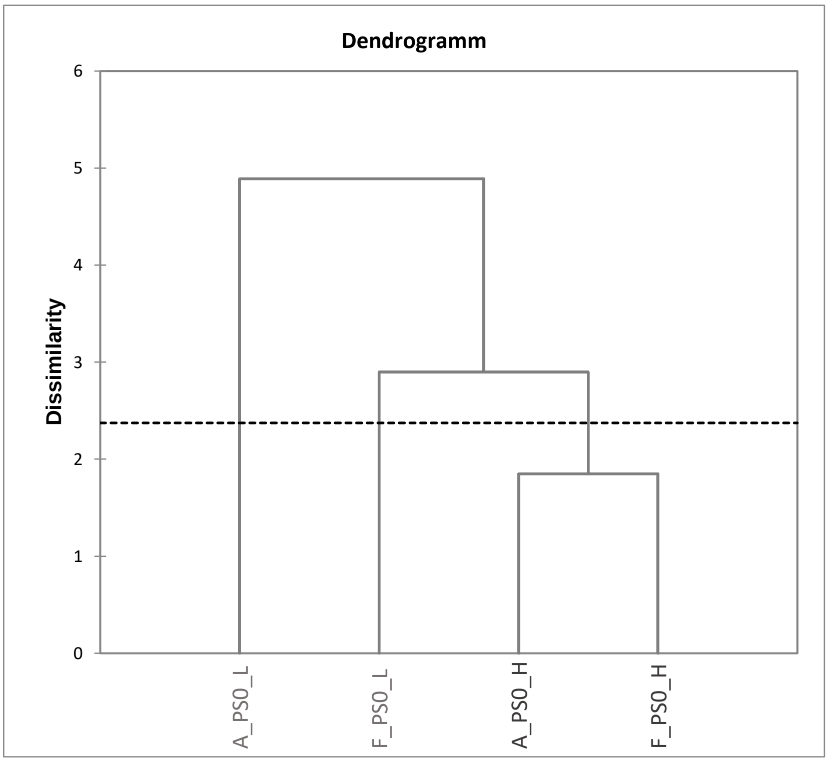

Figure S2: Results from Cluster Analysis based on sensory data of samples from variations without pod storage (PS0), dried at 60 °C (L, light grey) and at 80 °C (H, dark grey) from locations A and F.
